# Silver Nanowires and Silanes in Hybrid Functionalization of Aramid Fabrics

**DOI:** 10.3390/molecules27061952

**Published:** 2022-03-17

**Authors:** Alicja Nejman, Anna Baranowska-Korczyc, Katarzyna Ranoszek-Soliwoda, Izabela Jasińska, Grzegorz Celichowski, Małgorzata Cieślak

**Affiliations:** 1 Łukasiewicz Research Network–Textile Research Institute, Brzezinska St. 5/15, 92-103 Lodz, Poland; alicja.nejman@iw.lukasiewicz.gov.pl (A.N.); anna.baranowska-korczyc@iw.lukasiewicz.gov.pl (A.B.-K.); izabela.jasinska@iw.lukasiewicz.gov.pl (I.J.); 2 Department of Materials Technology and Chemistry, Faculty of Chemistry, University of Lodz, Pomorska St. 163, 90-236 Lodz, Poland; katarzyna.soliwoda@chemia.uni.lodz.pl (K.R.-S.); grzegorz.celichowski@chemia.uni.lodz.pl (G.C.)

**Keywords:** *para*-aramid, *meta*-aramid, silver nanowires, plasma surface modification, polydopamine, surface free energy, fabric durability, UV protection, hydrophobicity, conductive fabric

## Abstract

New functionalization methods of *meta*- and *para*-aramid fabrics with silver nanowires (AgNWs) and two silanes (3-aminopropyltriethoxysilane (APTES)) and diethoxydimethylsilane (DEDMS) were developed: a one-step method (mixture) with AgNWs dispersed in the silane mixture and a two-step method (layer-by-layer) in which the silanes mixture was applied to the previously deposited AgNWs layer. The fabrics were pre-treated in a low-pressure air radio frequency (RF) plasma and subsequently coated with polydopamine. The modified fabrics acquired hydrophobic properties (contact angle Θ_W_ of 112–125°). The surface free energy for both modified fabrics was approximately 29 mJ/m^2^, while for reference, *meta*- and *para*-aramid fabrics have a free energy of 53 mJ/m^2^ and 40 mJ/m^2^, respectively. The electrical surface resistance (R_s_) was on the order of 10^2^ Ω and 10^4^ Ω for the two-step and one-step method, respectively. The electrical volume resistance (R_v_) for both modified fabrics was on the order of 10^2^ Ω. After UV irradiation, the Rs did not change for the two-step method, and for the one-step method, it increased to the order of 10^10^ Ω. The specific strength values were higher by 71% and 63% for the *meta*-aramid fabric and by 102% and 110% for the *para*-aramid fabric for the two-step and one-step method, respectively, compared to the unmodified fabrics after UV radiation.

## 1. Introduction

Due to their low density, high tensile strength, excellent thermal stability, high specific strength, and modulus of elasticity, aramid fibers are widely used in special clothes accessories and technical products [1]. Their weakness, however, is their poor resistance to UV radiation. Aramids absorb UV radiation in the range of 300 to 400 nm, which results in breaking of the intermolecular bonds in the polymer that causes deterioration of the mechanical properties [2,3]. Therefore, it is important to protect them against UV radiation such as by modification with nanosilver, which also provides antibacterial and antistatic properties. Such functionalization significantly extends the scope of their application, such as materials for thermal sensors in smart clothes, for the protection of electronic communication, and in aviation [4,5,6]. The molecular structure of aramid fibers, which consist of aromatic rings and amide groups, makes them highly crystalline [7,8]. Due to the smooth and chemically inert surface, they show poor adhesion to modifiers. In order to improve the functionalization efficiency, an initial surface modification is performed through plasma activation and deposition of a polydopamine thin film by oxidative polymerization of dopamine [9]. The activation increases the free surface energy of aramid fibers and creates more reactive sites, enabling better binding between the polydopamine and the aramid surface [9,10,11]. Polydopamine is a biopolymer that forms a coating on the fiber surface through induced dopamine oxidative self-polymerization. This process enables the creation of carboxyl, amine, and catechol functional groups in order to strengthen the bond between the fiber and the modifier and improve the physical properties of the composites [12,13,14]. Polydopamine shows adhesion for most types of inorganic and organic surfaces, including precious metals, oxides, polymers, semiconductors, and ceramics [13,15]. Studies on the adhesion mechanism of the polydopamine film show that functional groups create covalent and non-covalent connections with inorganic or organic materials [15,16,17,18]. This type of preliminary modification increases the deposition efficiency of silver nanoparticles and improves the durability of the coating [9,19,20,21]. Silver nanowires (AgNWs) are one type of nanomodifier used for the multifunctionalization of textile materials. They have two UV absorption peaks; the peak at 350 nm can be attributed to surface plasmon resonance (SPR) along the silver nanowires, and the peak at about 380 nm can be considered the transverse direction of the SPR [22,23]. AgNWs absorb UV radiation, constituting a protective barrier for the fibrous structure covered with them, but they can also degrade. Air humidity has a significant influence on the degradation under the influence of UV radiation [24]. For higher air humidity, the oxygen and water contained in the air under the influence of UV radiation can create ozone and hydroxyl groups, which can initiate oxidation, hydrolysis, and photodegradation processes of the AgNWs [24,25]. For protection against UV radiation, silanes are used [26,27,28,29] because they have the ability to reflect UV radiation [28]. Hydrophobic silane-based textile materials are usually produced by surface treatment with mixtures of silane agents and silane cross-linkers [28,30]. One of the silanes used for the modification is 3-aminopropyltriethoxysilane (APTES), which contains a polar and chemically active aminopropyl group and three easily hydrolysable alkoxy groups. The hybrid organo-silica film obtained by APTES hydrolysis is hydrophilic, and the water contact angle measured by Zeng et al. [31] for the APTES film was 50°. The addition of diethoxydimethylsilane (DEDMS) to tetraethoxysilane (TEOS) before the hydrolysis increased the hydrophobicity of the obtained coatings was studied by Zhang et al. [32]. Souza et al. [33], for a mixture of APTES with (3-glycidoxypropyl) trimethoxysilane (GPTMS) (ratio of 2:1), obtained a coating with a water contact angle of 109.7°. Hasanzadeh et al. [34] coated a polyester-viscose fabric with silica nanoparticles prepared from TEOS to obtain a rough and hydrophobic surface. The fabric was then treated with functionalized polydimethylsiloxane (PDMS) to reduce the surface free energy. This fabric became hydrophobic with a contact angle of 151°. APTES is most often used as a stabilizer and a fastener to immobilize silver nanoparticles on the fiber surface [35].

In this work, *meta*- and *para*-aramid fabrics were modified with AgNWs and a mixture of silanes, APTES, and DEDMS. There were two methods of modification tested, a one-step method using a mixture of silanes with a AgNWs colloid (ratio of 1:10) and a two-step method (layer-by-layer), in which a mixture of silanes was applied on the AgNWs coating. The aim of the research was to protect aramid fabrics against UV radiation, improve the durability, conductivity, and hydrophobic properties, and assess which modification method is more effective. 

Obtained results will have a significant impact on the development of knowledge in the field of material engineering, surface physico-chemistry, and functionalization of aramid materials. The functionalization of aramid textile materials with AgNWs and silanes is an original strategy of improving functionality of aramid fabrics, by giving them mechanical resistance to UV radiation, hydrophobicity, and conductivity. Protection against UV radiation extends the usage of modified aramid fabrics, which has a beneficial effect on the environment. In addition, hydrophobicity reduces the contamination, including biofouling, and protects AgNWs against corrosion. In turn, imparting conductive properties aims to create conductive paths between elements and to protect electronic components against electrostatic discharge (ESD) in textronic products. Thanks to the combination of the functional properties of the AgNWs, silanes, and aramids, we developed modified aramid fabrics, which can be used in many fields, e.g., in the automotive and military industry, in protective clothing in high-risk occupations, to monitor vital functions or the level of available oxygen, and also in everyday life as textile materials for tents, umbrellas, and covering fabrics, etc.

## 2. Materials and Methods

### 2.1. Materials

*Meta*-aramid (poly (isophthalates-1,3-fenylodiamid)) (*m*Ar) fabric with a plain-weave and mass per unit area of 205 g/m^2^ made of 25 × 2 tex yarn (number of threads: in warp—230/10 cm, in weft—160/10 cm), (Newstar^®^, Yantai Tayho Advanced Materials Co., Ltd., Yantai, China) and *para*-aramid (poly (1,4-terephthalate-fenylodiamid)) (*p*Ar) fabric with a plain-weave and mass per unit area of 165 g/m^2^ made of 20 × 2 tex yarn (number of threads: in warp—240/10 cm, in weft—150/10 cm), (Kevlar^®^, DuPont, Londonderry, UK) were studied. Both fabrics were made in Łukasiewicz Research Network-Textile Research Institute using the harness loom (CCI Tech. Inc., New Taipei City, Taiwan).

### 2.2. Methods

#### 2.2.1. Functionalization Methods

##### Preparation of Silver Nanowires (AgNWs) Colloid 

The synthesis of AgNWs was described in a previous work [36]. In brief, AgNWs were synthesized by reduction of silver nitrate (AgNO_3_, 99.9%, Sigma Aldrich, Gillingham, UK) with ethylene glycol (C_2_H_6_O_2_, Avantor, Gliwice, Poland) in the presence of polyvinylpyrrolidone (PVP, 55,000 g/mol, Sigma Aldrich, UK).

The synthesis was performed in a Radleys 1l Reactor-Ready (Radleys, Essex, United Kingdom) reactor equipped with a mechanical stirrer, working at a speed of 150 RPM. The reactor has a double heating jacket, allowing for precise temperature control performed by an external thermostat during the entire synthesis. The temperature was set at 170 ± 2 °C.

A total of 600 mL of ethylene glycol, 30 g of PVP, and 0.42 g of sodium chloride (NaCl, CHEMPUR, Piekary Śląskie, Poland) were heated to 167 °C (constant temperature control) and stirred at 150 RPM to obtain a homogenous solution. Then, a mixture of 6.12 g of AgNO_3_ in 300 mL of ethylene glycol was added with a peristaltic pump at a rate of 3.33 mL/min for 90 min into the heated solution of ethylene glycol, PVP, and NaCl. When the silver precursor solution was added, the colloid was stirred at 150 RPM and heated for 60 min at 167 °C.

After the synthesis, the solution was air-cooled to about 25 °C. To remove ethylene glycol and excess PVP from the colloid, the subsequent, small parts of the solution were diluted with acetone (C_3_H_6_O, 99,5%, Avantor, Gliwice, Poland) with a ratio of 1:10 and shaken for 10 min. Then, the silver nanowires were dispersed in anhydrous ethyl alcohol (C_2_H_6_O, 99,8%, Avantor, Gliwice, Poland). The obtained AgNWs colloid concentration was 4000 ppm, the length of the AgNWs was about 10 ± 2 µm, and the diameter was about 42 ± 3 nm. 

##### Low-Pressure Air RF Plasma Treatment of Aramid Fabrics 

*Para*-aramid and *meta*-aramid fabrics were washed in diethyl ether for 30 min and dried at 25 °C. Next, both fabrics were treated with a low-pressure air RF plasma (Zepto plasma system, Model 2. Diener Electronic, Ebhausen, Germany) for 10 min. The pressure in the plasma chamber was set at 0.3 mbar with laboratory air (Linde Gaz, Kraków, Poland) with the purity: 20% of O_2_ and 80% of N_2_ (40% relative humidity) as the working gas. The power of the plasma was set at 50 W and the generator frequencies was 40 kHz.

##### Polydopamine Functionalization of Aramid Fabrics

**Figure 1:**
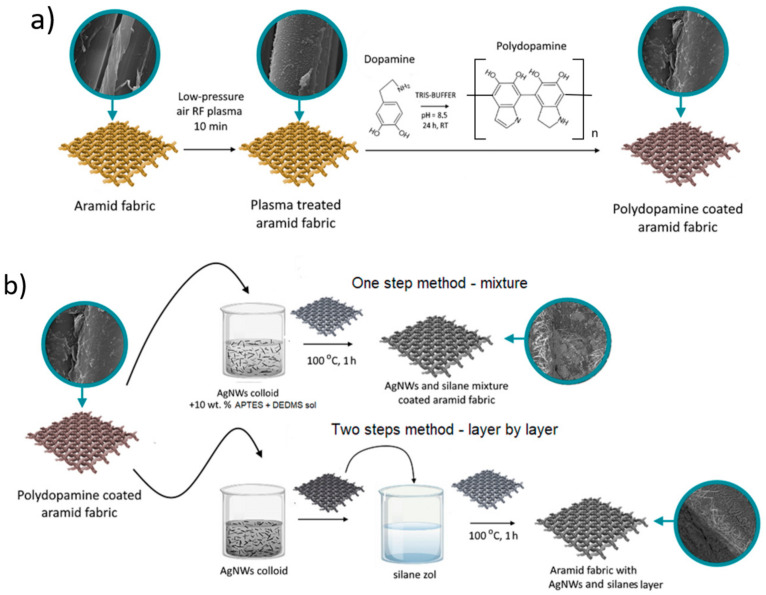
The scheme of the modification of aramid fabrics with (**a**) low-pressure air RF plasma and polydopamine and (**b**) the application of AgNWs and silanes by the mixture and layer-by-layer method.

A dopamine solution with a concentration of 2 g/L was prepared by dissolving dopamine hydrochloride (Sigma Aldrich, UK) in distilled water. The pH of the solution was adjusted to 8.3 by adding tris/glicyne buffer (BIO RAD, Warszawa, Poland). The plasma treated aramid fabrics were immersed in the dopamine solution for 24 h (Figure 1a). Under the same conditions, a polydopamine coating was prepared on the surface of the aluminum plate. This material was used for the acquisition of a reference FTIR spectrum of pure polydopamine. After that, all samples were rinsed with distilled water three times and then dried for 24 h at 25 °C.

##### Silanes Sol Preparation

APTES (Unisil, Tarnów, Poland) and DEDMS (Sigma Aldrich, Gillingham, United Kingdom) differ in their chemical structure, brittleness, and hydrophobicity. To prepare the AgNWs and silane modified aramid fabrics, silanes sol was prepared by mixing 15.00 g of APTES, 1.12 g of DEDMS, and 3.93 g of 1 M HCl (Avantor, Gliwice, Poland). The silanes mixture was shaken for 30 min. Next, ethyl alcohol (C_2_H_6_O, 99.8%, Avantor, Gliwice, Poland) was added at 50% by volume of the silanes mixture.

##### AgNWs and Silanes Functionalization of Aramid Fabrics

The one-step (mixture) and two-step (layer-by-layer) methods were used for the functionalization of aramid fabrics with AgNWs and silanes (Figure 1b). In the one-step method, aramid fabrics were 5-fold dipped in a mixture of a AgNWs colloid (Ag) with 10 wt.% silanes sol (S) for 1 min. After the mixture was applied every time, the fabrics were dried in an oven at 100 °C for 1 h. The fabrics modified with the mixture (Ag + S) are denoted as *m*Ar/RF/PD/5Ag+S and *p*Ar/RF/PD/5Ag+S for the *meta*- and *para*-aramid fabric, respectively.

In the two-step method, fabrics were dipped in the AgNWs colloid for 1 min and dried at 25 °C. The AgNWs colloid (Ag) was applied 5 times. Then, the fabrics were dipped 1 time in the silanes sol (S) and then dried in an oven at 100 °C for 1 h. The fabrics modified with the layer-by-layer (Ag/S) method are denoted as *m*Ar/RF/PD/5Ag/S and *p*Ar/RF/PD/5Ag/S for the *meta*- and *para*-aramid fabric, respectively.

Moreover, as control samples, aramid fabrics were modified with AgNWs. A 1-fold (*m*Ar/RF/PD/1Ag and *p*Ar/RF/PD/1Ag) and 5-fold (*m*Ar/RF/PD/5Ag and *p*Ar/RF/PD/5Ag) colloid application was used.

In Table 1, the values of mass per unit area of the reference and functionalized aramid fabrics are summarized.

##### UV Irradiation

The UV irradiated unmodified and modified fabrics were placed between two (100 W) LED diodes with the maximum wavelength of the emitted light at 365 nm for 96 h. The irradiance (W) was determined by using the CUV 4 irradiance sensor (Kipp & Zonen B.V., Delft, Netherlands) to measure the intensity of the light in the UV range (305–385 nm). The supplied irradiation energy (P) was calculated from Equation (1) [37] and determined to be 46,719 kJ/m^2^ for one LED diode.

(1) $$P=\frac{t\cdot 3.6\cdot \Sigma W}{n}$$

where P is the irradiation energy (kJ/m^2^), t is the irradiation time (h), W is the irradiance (W/m^2^), and n is the number of measurements during the irradiation time t.

#### 2.2.2. Characterization Methods

##### SEM/EDS Analysis

The morphology and chemical composition analysis were performed using a scanning electron microscope (SEM) (Nova NanoSEM 450 FEI, Hillsboro, OR, USA) and VEGA 3 (TESCAN, Brno, Czech Republic) with an energy dispersive spectroscopy (EDS) X-ray microanalyzer INCA Energy (Oxford Instruments Analytical, High Wycombe, United Kingdom) with a magnification of 2500×, 10,000×, and 20,000×. A total of three EDS spectra were recorded for each sample, and mean values of the weight percentage of the elements were determined.

##### Optical Microscopy

The images of the fabric surface before and after the abrasion process were acquired using an optical microscope DSX1000 (OLYMPUS, Tokyo, Japan) and using the DEPH function to increase the depth of field. A magnification of 300× was used for all images.

##### FTIR Analysis

FTIR/ATR (Fourier transform infrared spectroscopy/attenuated total reflectance) spectra of reference and plasma-treated yarns were recorded in the range 600–4000 cm^−1^ using Nicolet IS 50 spectrometer (Thermo Fisher Scientific Inc., Bartlesville, OK, USA) with GATR (grazing angle attenuated total reflectance) accessory (Harrick Scientific Products Inc., New York, NY, USA) using the MCT (mercury-cadmium-telluride) detector. This accessory allows to collecting spectra from the surface of the fibers at a depth of about up to 50 nm.

##### Surface Properties

The analysis of the surface properties of the fabrics was performed by the goniometric method using the goniometer PGX (Fibro System AB, Stockholm, Sweden). In order to determine the surface free energy (γ_s_), three standard liquids with known surface tensions and different values of dispersive and polar components were applied (Table 2). The drop of liquid with a volume of 4 µL was applied. A total of three repetitions for each sample were used. The surface free energy was determined according to the Wu model (Equation (2)), which is based on the assumptions of the Owens-Wendt model [7,38,39,40,41,42] but describes the intermolecular interaction using the harmonic mean.

γ_l_(1+cosθ) = 4((γ_l_^d^γ_s_^d^)/γ_l_^d^ + γ_s_^d^ + γ_l_^p^γ_s_^p^/γ_l_^p^ + γ_s_^p^) and γ_s_ = γ_s_^d^ + γ_s_^p^ (2)

where γ_s_^d^ and γ_s_^p^ are the dispersive and polar components of a solid, respectively, and γ_l_^d^ and γ_l_^p^ are the dispersive and polar components of a liquid, respectively.

##### Specific Strength

The study of the specific strength of the fabrics before and after UV irradiation was performed by using the Instron 3367 Test Machine (United Kingdom) in accordance with PN-EN ISO 13934-1:2013-07 Textiles—Tensile properties of flat products—Part 1: Determination of maximum force and relative elongation at maximum force by the strip method. A total of five repetitions for each sample were used. Samples were conditioned for 24 h at a temperature of 21.0 ± 1.0 °C and a relative humidity (RH) of 64.4 ± 0.1% and then tested in the same conditions.

##### Abrasion Resistance

The resistance to abrasion of the fabrics before and after UV irradiation was studied using the Martindale unit (United Kingdom) in accordance with PN-EN ISO 12947-2:2017-02 Textiles—Determination of the abrasion resistance of fabrics by Martindale method—Part 2: Determination of specimen breakdown. A total of three repetitions for each sample were used. Samples were conditioned for 24 h at a temperature of 21.1 ± 0.1 °C and an RH of 64.4 ± 0.1% and were tested in the same conditions. A standard woolen fabric was used as an abrasive cloth. An abrasive load of 12 kPa was applied.

##### Conductivity

The electrical surface resistance (R_s_) and electrical volume resistance (R_v_) of the fabrics before and after UV irradiation were determined according to the PN-EN 1149-1:2008 for R_s_ and PN-EN 1149-2:1999+A1:2001 for R_v_, using a set of standard electrodes and a 6206 teraohmmeter (ELTEX, Weil am Rhein, Germany). The fabrics were conditioned for 24 h in the HCZ 0030 L(M) chamber (Heraeus, Hanau, Germany) at a temperature of 23.0 ± 2.0 °C and an RH of 25.0 ± 5.0% and were tested in the same conditions. 

## 3. Results and Discussion

### 3.1. Modification of the Aramid Fabrics with Low-Pressure Air RF Plasma and Polydopamine

**Figure 2:**
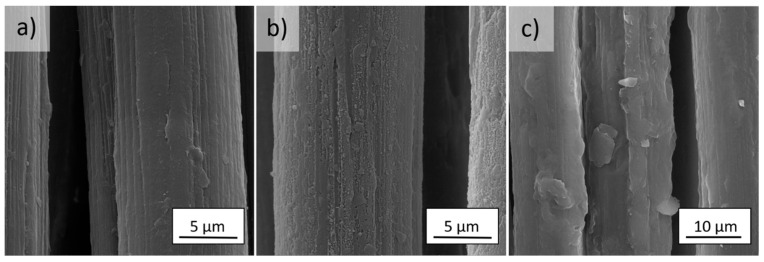
SEM images of (**a**) reference, (**b**) plasma treated, and (**c**) polydopamine modified *m*Ar fibers (magnification of 10,000×).

The surface of the *meta*- (Figure 2a) and *para*-aramid (Figure 2a) fibers is smooth with visible longitudinal cracks. Aramid fibers have a fibrillar structure. Activation in low-pressure RF plasma causes an increase in the unevenness and surface roughness (Figure 1b and Figure 2b).

**Figure 3:**
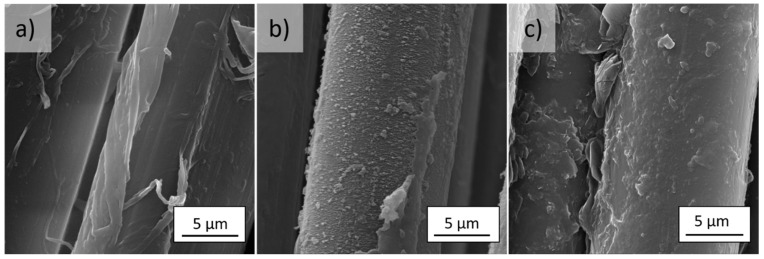
SEM images of (**a**) reference, (**b**) plasma treated, and (**c**) polydopamine modified *p*Ar fibers (magnification of 10,000×).

The dopamine solution prepared for the functionalization of the plasma-activated aramid fabrics was colorless and transparent. During the oxidative autopolymerization of dopamine, the color of the solution quickly turned pink as catechol was oxidized to benzoquinone. Then, the pink solution slowly turned to dark brown. This indicates that after polymerization, the reaction of melanin formation takes place, resulting in the creation of polydopamine (PD) with a high strength of irreversible covalent bonds on the matrix. After modification, polydopamine formed a layer on the surface of both types of fibers (Figure 2c and Figure 3c).

**Figure 4:**
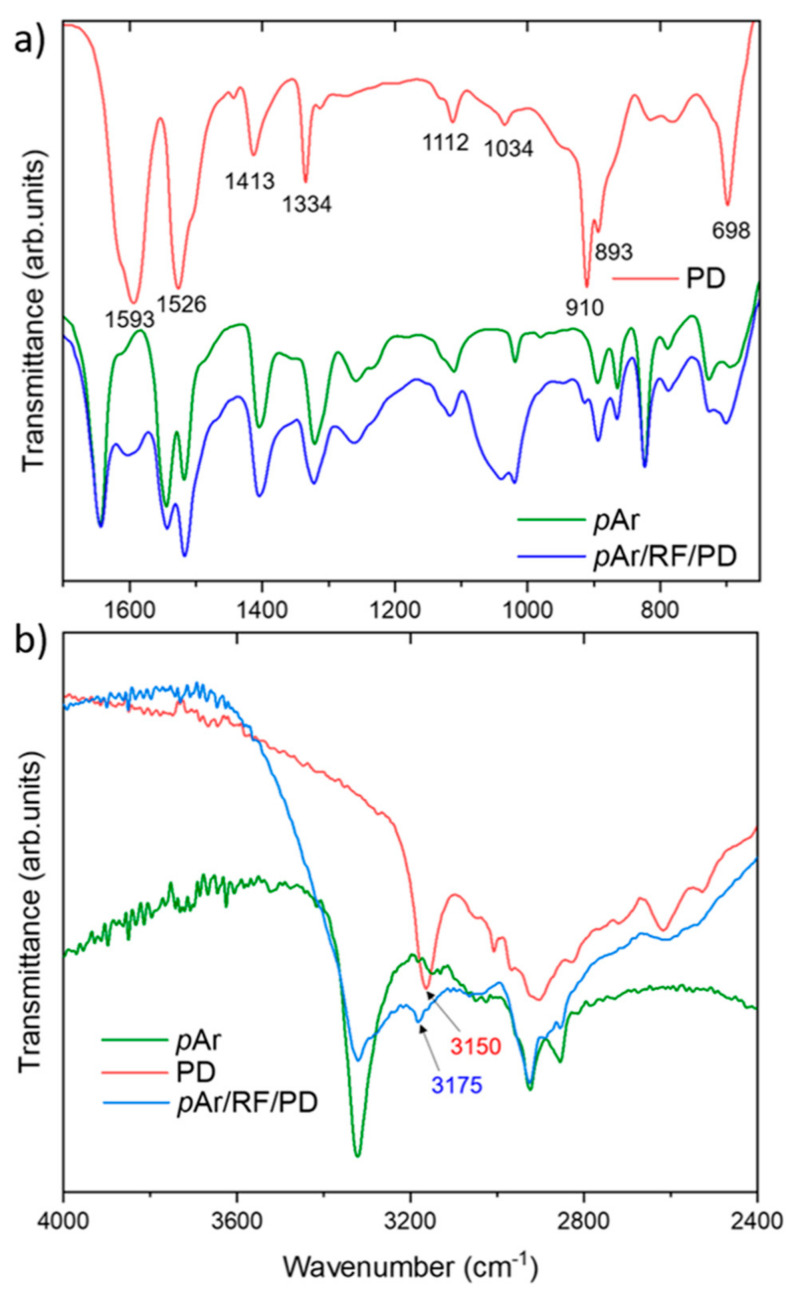
FTIR spectrum of polydopamine, reference, and RF plasma and polydopamine functionalized *para*-aramid fabrics in the range of (**a**) 600–1750 cm^−1^ and (**b**) 2400–4000 cm^−1^.

The presence of the polydopamine coating on the aramid fiber surface was observed during the SEM analysis and confirmed by the FTIR spectroscopy results (Figure 4). In Table 3, the characteristic bands and their wavenumbers for the *p*Ar fabric and polydopamine are presented. After application of the dopamine coating on the *p*Ar fabric, an increase in the band intensity at 698 cm^−1^, corresponding to the C–H bond out-of-plane of the substituted aromatic ring, and at 1513 cm^−1^, corresponding to the C=N bond stretching vibrations of the aromatic ring, in relation to the unmodified *p*Ar (Figure 3a) is observed. In the spectrum for the *p*Ar/RF/PD fabric, bands are present in the spectrum for polydopamine but not in the spectrum for the reference *p*Ar fabric. These are the bands at 911 cm^−1^ characteristic of the bending vibrations of the CONH bond and CN stretching, at 1440 cm^−1^ corresponding to the stretching vibrations of the C=C bond of the aromatic ring (Figure 4a), and at 3175 cm^−1^ corresponding to the stretching vibrations of the N–H bond (Figure 4b).

### 3.2. AgNWs and Silanes Modified Aramid Fabrics before and after UV Irradiation 

**Figure 5:**
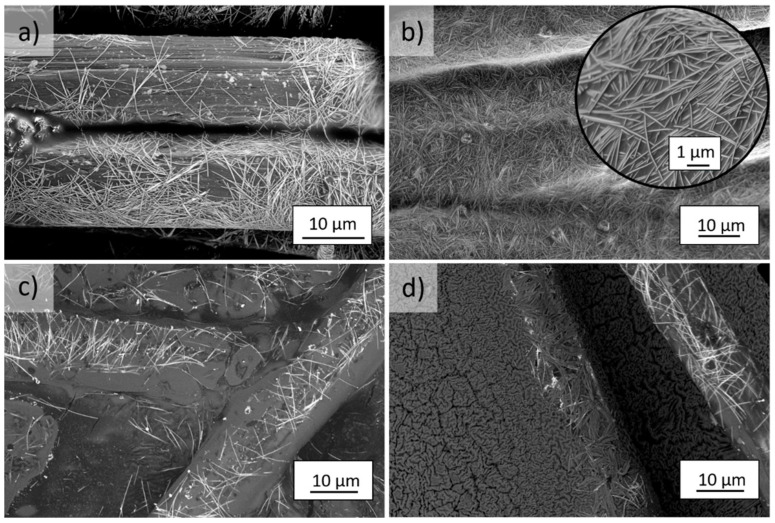
SEM images of modified *m*Ar fibers after the (**a**) 1-fold and (**b**) 5-fold application of AgNWs and the (**c**) mixture Ag+S, and (**d**) layer-by-layer Ag/S modification (magnification of 2500× and 20,000×).

**Figure 6:**
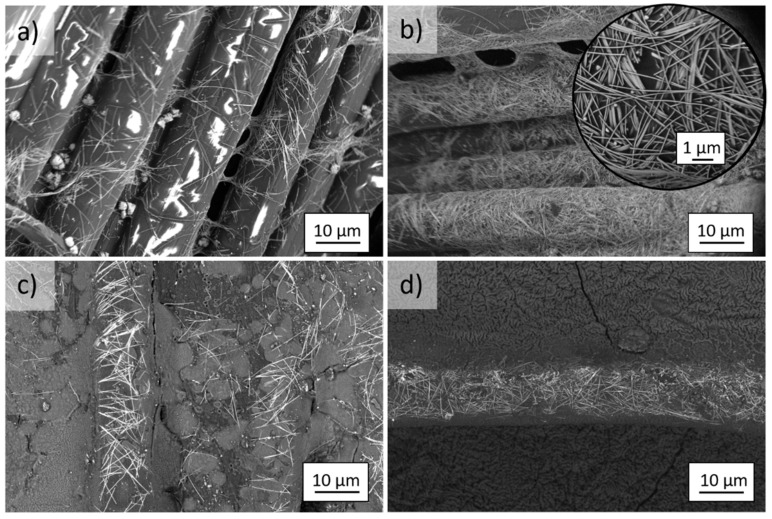
SEM images of modified *p*Ar fibers after the (**a**) 1-fold and (**b**) 5-fold application of AgNWs and the (**c**) mixture Ag+S, and (**d**) layer-by-layer Ag/S modification (magnification of 2500× and 20,000×).

SEM analysis shows that the surface of both aramid fibers after the 1-fold application of AgNWs (*m*Ar/RF/PD/1Ag and *p*Ar/RF/PD/1Ag) is covered unevenly (Figure 5a and Figure 6a). After the 5-fold application, the coating is uniform (Figure 4b), with few areas without AgNWs (Figure 6b). AgNWs are found both on the surface of the fibers and inside the fabric structure. After application of the Ag+S mixture (Figure 5c and Figure 6c), the AgNWs were embedded inside the silanes coating and deposited both on the surface of the fibers and in the spaces between them. In the case of the layer-by-layer method (Figure 5d and Figure 6d), a layer of silanes covers the surface of the fabric with AgNWs. There are also fibers with AgNWs that protrude above the silanes surface.

EDX analysis of the *m*Ar and *p*Ar fabric shows the presence of C, N, and O (Table 4). For the *m*Ar fabric, the content of C, N, and O is 68, 11, and 20 wt.%, respectively. For the *m*Ar/RF/PD/1Ag fabric, the content of Ag is 3 wt.%, and for *m*Ar/RF/PD/5Ag, it is 6-fold higher (Table 4). With the increase in the multiplicity of the AgNWs application, the content of C, N, and O decreased, which proves the effective coverage of the aramid fibers with AgNWs. For *m*Ar/RF/PD/5Ag+S, the C and N content does not change significantly, and the O content, which is derived from the ethoxyl groups in silanes, increases by about 50% compared to *m*Ar/RF/PD/5Ag. A decrease in the C content by 26% and an increase in the O and N content by 70% and 24%, respectively, are noted for *m*Ar/RF/PD/5Ag/S in relation to *m*Ar/RF/PD/5Ag. These changes are due to the presence of the silanes layer, which contains ethoxy groups and an amino group. The Ag content on the fiber surface is about 60% and 30% lower for *m*Ar/RF/PD/5Ag+S and *m*Ar/RF/PD/5Ag/S compared with *m*Ar/RF/PD/5Ag. The Si content is 5 wt.% and 7 wt.% for *m*Ar/RF/PD/5Ag+S and *m*Ar/RF/PD/5Ag/S, respectively.

The content of C, N, and O for the *p*Ar fabric is 66, 13, and 19 wt.%, respectively. In the case of *p*Ar/RF/PD/1Ag, the Ag content is 6 wt.%, and after the 5-fold application, it is 3.5 times higher. The content of C, N, and O decreases with the increase in the number of AgNWs applications, which indicates the effective coverage of the fibers with AgNWs. For *p*Ar/RF/PD/5Ag+S, the C and N content stays at the same level in relation to *p*Ar/RF/PD/5Ag. The O content increases by 48%, which is due to the presence of ethoxyl groups in the silanes. For *p*Ar/RF/PD/5Ag/S, the C content is lower by 22%, and the N and O content is higher by 46% and 72%, respectively, which, similarly to the *meta*-aramid fabric, is due to the surface being covered with silanes. For *p*Ar/RF/PD/5Ag+S and *p*Ar/RF/PD/5Ag/S, the Ag content on the fiber surface is lower by about 64% and 42%, respectively, than for *p*Ar/RF/PD/5Ag. The Si content is 5 wt.% for *p*A/RF/PD/5Ag+S and 6 wt.% for *p*Ar/RF/PD/5Ag/S. 

**Figure 7:**
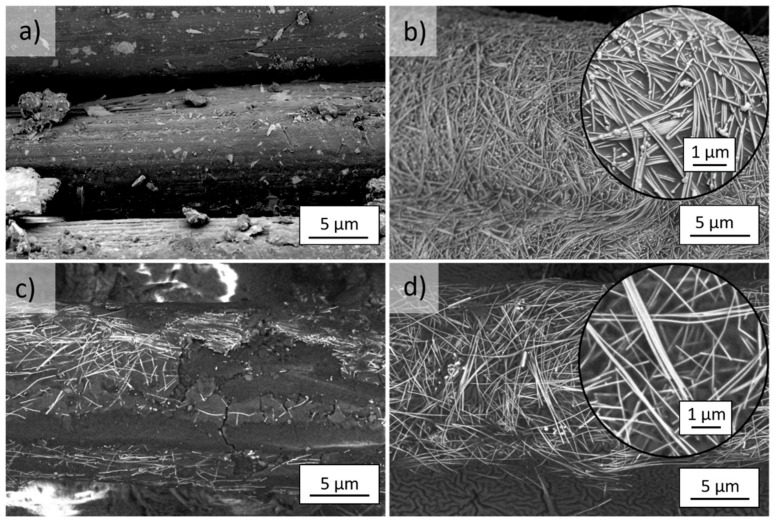
SEM images of UV irradiated (365 nm, 96 h) *m*Ar fibers (**a**) reference and after (**b**) 5-fold AgNWs application, (**c**) mixture Ag+S and (**d**) layer by layer Ag/S modification (magnification of 6500× and 20,000×).

**Figure 8:**
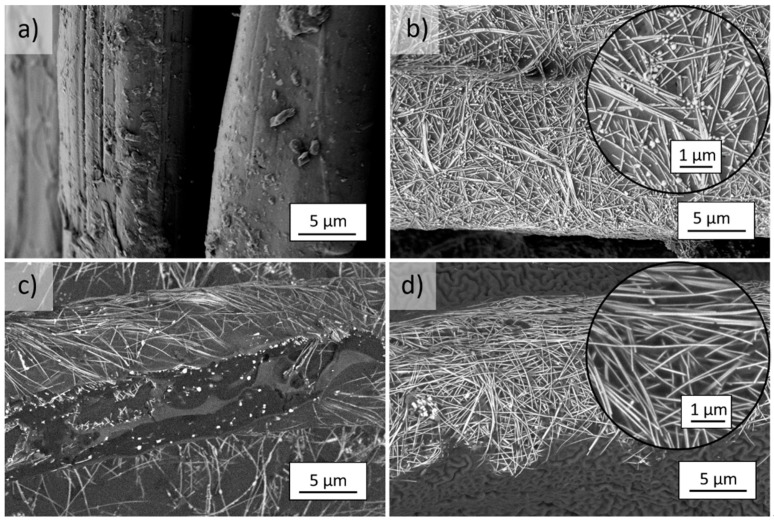
SEM images of UV irradiated (365 nm, 96 h) *p*Ar fibers (**a**) reference and after (**b**) 5-fold AgNWs application, (**c**) mixture Ag+S, and (**d**) layer-by-layer Ag/S modification (magnification of 6500× and 20,000×).

After 96 h of UV irradiation of the unmodified *m*Ar (Figure 7a) and *p*Ar (Figure 8a) fabrics, the roughness of the fibers surface increased, and polymer fragments were present on the surface. The UV irradiation caused degradation of the AgNWs on the fabrics, as evidenced by silver precipitates on their surface (Figure 7b insert, Figure 8b insert). The coating on the *m*Ar and *p*Ar fibers modified with the Ag + S mixture was cracked (Figure 7c and Figure 8c), and coating defects were visible, which proves that fragments of the Ag + S coating were detached (Figure 7c). UV radiation causes breaking of the polysiloxane bonds and photo-oxidation, which induces the formation of silanol and carbonyl groups [27]. In the case of the layer-by-layer method, the AgNWs did not degrade and did not change their morphology (Figure 7d and Figure 8d). In the mixture method, the AgNWs were more exposed to the UV radiation due to the thin coating of silanes compared with the layer-by-layer method. This may cause the degradation of AgNWs and damages in silanes coating.

### 3.3. Surface Properties 

Table 5 presents the values of contact angles for water (Θ_W_), formamide (Θ_F_), and diiodomethane (Θ_DIM_) for the unmodified and modified fabrics.

For the *m*Ar fabric, the Θ_W_ value is 64°, and after plasma activation, it decreases by 70%. For the *p*Ar fabric, the Θ_W_ value is 20% higher than for the *m*Ar fabric. Plasma treatment reduced the Θ_W_ value by 84%. The decrease in the Θ_W_ value of *p*Ar/RF is greater than for *m*Ar/RF because of the chemical structure of both aramids and the higher degree of crystallinity of *para*-aramid, whose structure is more rigid and orderly, which causes greater changes on the fibers surface as a result of plasma etching [7]. After the application of the polydopamine coating, for the *m*Ar/RF/PD and *p*Ar/RF/PD fabrics, the value of Θ_W_ is 0°. The decrease in the Θ_W_ value is due to the presence of active polar functional groups on the surface, such as –OH, –NH_2_, or –NH (Figure 4), which interact with the polar liquid. A decrease in the Θ_W_ value was also observed by Sabdin et al. [42], who modified polypropylene (PP) meshes by immersing them in 10 mM dopamine buffered with TRIS at pH 8.5 for 24 h. Then, the PP meshes were washed several times with distilled water and dried at 40 °C. The Θ_W_ value decreased from 138.9° to 74.1°. Jiang et al. [43] immersed a PP membrane for 24 h in a 1.0 g/L aqueous dopamine solution prepared by dissolving 0.25 g of dopamine in 250 mL of deionized water. Then, 1 mL of 1 M NaOH was added to the solution to adjust the pH to 9. The Θ_W_ value decreased from 117.5° to 54.3°. Chen et al. [44] observed a decrease in the Θ_W_ value from 143.7° to 134.2° after modification of the PP nonwoven fabric in an aqueous solution of dopamine hydrochloride at a concentration of 2 g/L and buffered with TRIS (pH = 8.5) for 24 h at 60 °C and then the samples were washed four times with ultrasounds and dried in a vacuum at 60 °C. This indicates the exposure of hydroxyl and amine groups in polydopamine on the nonwoven surface, which causes the increase in the wettability. 

The Θ_W_ values for both aramid fabrics with AgNWs are comparable and higher than for the unmodified fabrics, and their hydrophobicity increases with the increasing number of AgNWs deposited.

Fabrics modified with the mixture and layer-by-layer methods are hydrophobic. The *m*Ar/RF/PD/5Ag+S and *p*Ar/RF/PD/5Ag+S fabrics have the highest Θ_W_ values, which are 125° and 120°, respectively. For the *m*Ar/RF/PD/5Ag/S and *p*Ar/RF/PD/5Ag/S fabrics, the Θ_W_ values are 112° and 114°, respectively. Zeng et al. [31] reported that the water contact angle of the APTES film is 52°, which means that APTES is hydrophilic. In turn, Zhang et al. [32] measured the contact angle of a DEDMS/TEOS silica aerogel (ratio of 0.26), which was 139.9°, while TEOS is hydrophilic [45].

**Figure 9:**
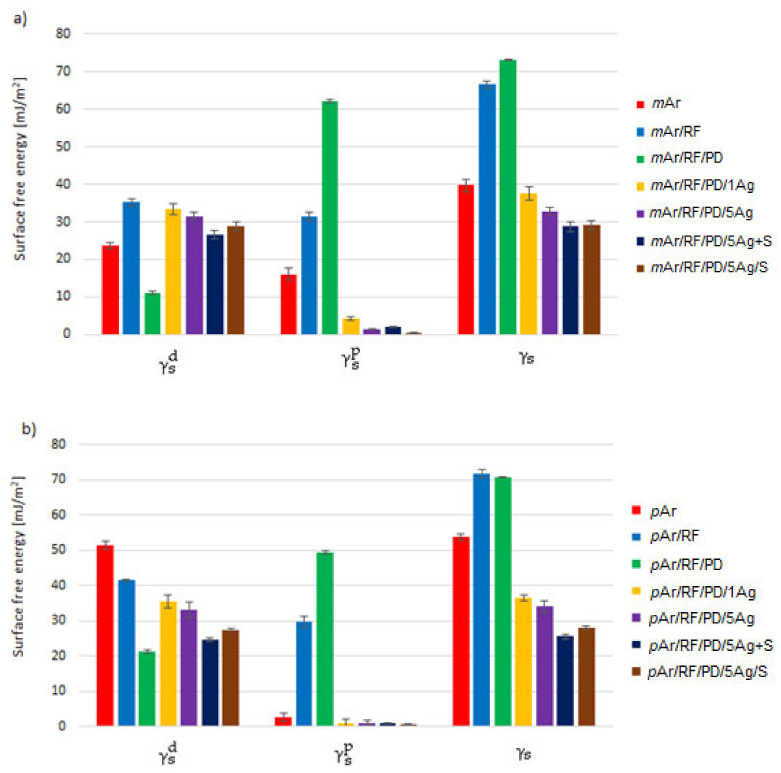
Surface free energy (γ_s_) of unmodified and modified *m*Ar (**a**) and *p*Ar (**b**) fabrics (γ_s_^d^—dispersive component, γ_s_^p^—polar component).

The value of the surface free energy (γ_s_) for the *m*Ar fabric is 39.8 mJ/m^2^, which is lower than for the *p*Ar fabric by about 35% (Figure 9a,b). Plasma treatment and modification with polydopamine resulted in a significant increase in the γ_s_ values by about 68% and 84%, respectively, for *m*Ar and by about 87% and 83% for *p*Ar fabrics in relation to the unmodified fabrics. After the 1- and 5-fold AgNWs application, the γ_s_ value slightly decreased with the increase in the modification multiplicity for both types of aramid fabrics and is slightly lower in relation to the unmodified fabrics. The γ_s_ values for the aramid fabrics modified with silanes by both methods are a little lower than for the fabrics after the 5-fold AgNWs application.

The dispersion component value (γ_s_^d^) of the *m*Ar fabric is 23.8 mJ/m^2^, which is lower than that of the *p*Ar fabric by about 50%. The plasma treatment causes a 52% increase and 21% decrease in the γ_s_^d^ component for the *m*Ar/RF and *p*Ar/RF fabric, respectively. The decrease in γ_s_^d^ for the *p*Ar fabric after plasma treatment in comparison with the *m*Ar fabric is related to the higher degree of crystallinity and the fibrillization phenomenon of the reference *p*Ar fabric (Figure 3a). For *m*Ar fabric surface, the enlargement of the surface roughness is observed after plasma treatment (Figure 2b), which causes the increase in the γ_s_^d^ value. After modification with polydopamine, the value of the γ_s_^d^ component decreases in relation to the unmodified fabrics as result of the formation of a layer of polydopamine on the surface of the fibers (Figure 2c and Figure 3c). For both fabrics, after the 1-fold AgNWs application, the γ_s_^d^ values are comparable. The lower γ_s_^d^ value for *p*Ar/RF/PD/1Ag compared with the reference fabric is caused by the fibrillization process, which significantly affects the dispersion component value. For the 5-fold AgNWs modified fabrics, the γ_s_^d^ values are still comparable and lower than for the 1-fold AgNWs modification, which may be related to the smoothing of the discontinuous surface after successive AgNWs applications. For both aramid fabrics modified with silanes by the mixture and layer-by-layer method, the γ_s_^d^ values are lower in relation to the fabrics after the 5-fold AgNWs application. Slightly higher γ_s_^d^ values are found for the layer-by-layer method, which may be because of the visible AgNWs coated fibers that protrude above the smooth silane surface. 

The value of γ_s_^p^ for *m*Ar is 16.0 mJ/m^2^, which is six times higher than the value for *p*Ar. After plasma treatment, the γ_s_^p^ values increase and are comparable for both fabrics. The polydopamine application causes a further increase in the γ_s_^p^ values by a factor of 2 and 1.7 for *m*Ar/RF/PD and *p*Ar/RF/PD, respectively. For fabrics modified with silanes by both methods, the γ_s_^p^ values are lower compared to the unmodified fabrics. The values of the polar components are higher than the dispersive component values only for the fabrics modified with polydopamine. Unmodified fabrics have a smooth surface with numerous longitudinal cracks and fibrils. The surface is chemically inert, and there are no active functional groups. After application of polydopamine, an increase in γ_s_^p^ in relation to γ_s_^d^ is caused by an increase in the polarity of the polydopamine surface due to the presence of active functional groups on the fiber surface: –OH, –NH_2_, or –NH (Figure 4).

### 3.4. The Durability before and after UV Irradiation

#### 3.4.1. Specific Strength

**Figure 10:**
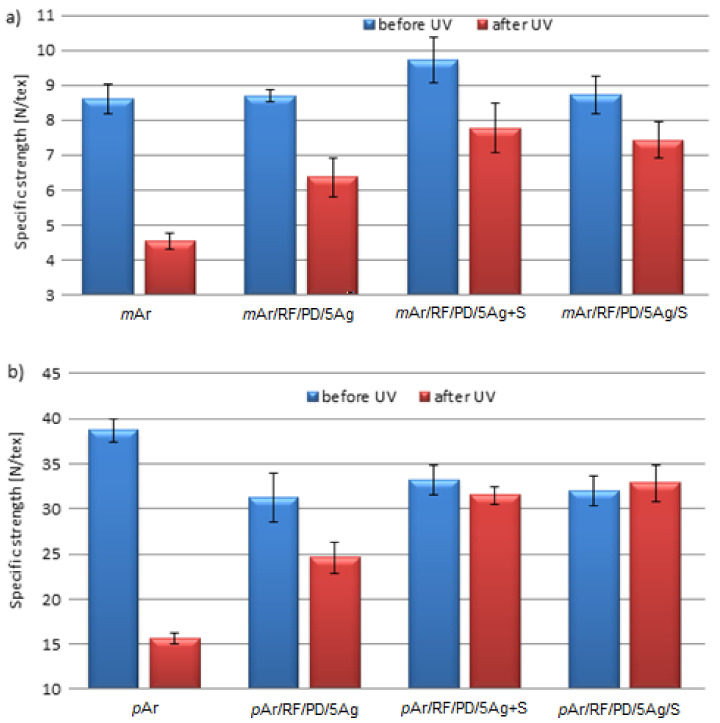
Specific strength of unmodified and modified *m*Ar (**a**) and *p*Ar (**b**) fabrics before and after UV irradiation (365 nm, 96 h).

The specific strength of the *m*Ar fabric (8.6 N/tex) (Figure 10a) is 4.5 times lower than that of the *p*Ar fabric, which does not change for *m*Ar/RF/PD/5Ag and *m*Ar/RF/PD/5Ag/S. For *m*Ar/RF/PD/5Ag+S, the specific strength is higher than for the *m*Ar fabric by about 12%. In the case of the modified *p*Ar fabrics, a decrease by about 19%, 14%, and 17% is observed for *p*Ar/RF/PD/5Ag, *p*Ar/RF/PD/5Ag+S, and *p*Ar/RF/PD/5Ag/S, respectively, compared to the unmodified fabric. The higher specific strength values after the application of the Ag+S mixture compared with the AgNWs modified fabrics are probably due to the stronger cross-linking of AgNWs with silanes, which occurred both on the surface of the fabric fibers and in the spaces between the fibers. For the layer-by-layer method, the cross-linking between the AgNWs and the silanes layer takes place only on the surface. The layer-by-layer modification does not change the specific strength of the fabrics in relation to the fabrics with AgNWs.

After 96 h of UV irradiation, an almost 50% decrease in the specific strength is observed for the *m*Ar fabric (Figure 10a). The specific strength is higher by 40%, 71%, and 63% for *m*Ar/RF/PD/5Ag, *m*Ar/RF/PD/5Ag+S, and *m*Ar/RF/PD/5Ag/S, respectively, compared with the *m*Ar fabric after UV irradiation. For the unmodified *p*Ar fabric, there is a 60% decrease in the specific strength. For *p*Ar/RF/PD/5Ag, *p*Ar/RF/PD/5Ag+S, and *p*Ar/RF/PD/5Ag/S (Figure 10b), their specific strength values are 58%, 102%, and 111% higher than for the UV irradiated *p*Ar fabric, respectively. The significantly higher specific strength values after the AgNWs and silanes mixture and layer-by-layer modification is related to the reflection of UV radiation by silanes, which protects the AgNWs and aramid from UV degradation. Shi et al. [28] also observed the effect of the silane layer on the reflectance of UV radiation for a cotton fabric modified with graphene oxide and a KH570 silane layer. Tragoonwichian et al. [29] modified a cotton fabric using vinyletriethoxysilane, which significantly reduced the transmittance of UV radiation through the fabric.

#### 3.4.2. Abrasion Resistance

**Figure 11:**
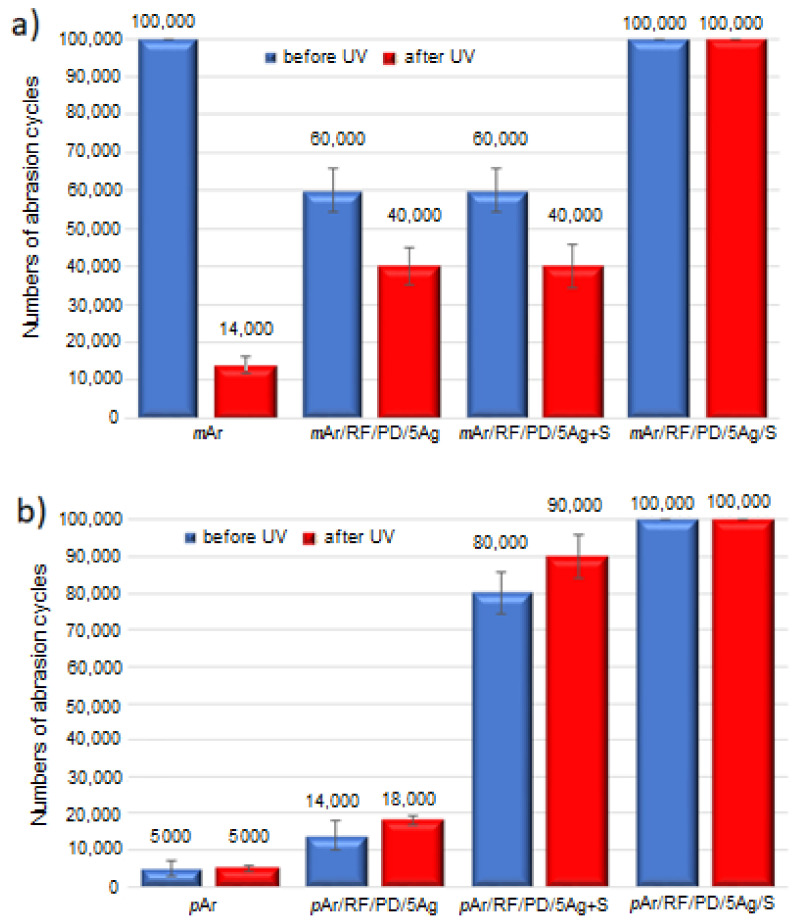
Results of the Martindale’s abrasion test for unmodified and modified *m*Ar (**a**) and *p*Ar (**b**) fabrics before and after UV irradiation (365 nm, 96 h).

The *m*Ar fabric is abrasion resistant. After 100,000 cycles (Figure 11a), no breakage of the threads is observed (Figure S1a, Supplementary Materials). For the *p*Ar fabric, thread abrasion (Figure S1b, Supplementary Materials) occurs after 5000 cycles (Figure 11b), probably due to the less ordered structure and lower degree of crystallinity of *m*Ar than of *p*Ar. For the *m*Ar/RF/PD/5Ag and *m*Ar/RF/PD/5Ag+S fabric, a decrease in the number of abrasion cycles by 40,000 compared to the *m*Ar fabric is observed, which proves that AgNWs reduce the abrasion resistance of *meta*-aramid. In the case of the *para*-aramid fabrics, the application of AgNWs and a mixture of AgNWs and silanes improves the abrasion resistance and increases the number of abrasion cycles (Figure 11b) by 7000 and 75,000, respectively. For the layer-by-layer method, no thread damage is observed for both fabrics after 100,000 abrasion cycles.

After UV exposure, the abrasion resistance of the mAr fabric decreased, the breakage of the threads is observed (Figure S1a, Supplementary Materials), and the number of abrasion cycles decreases by a factor of seven. For the *m*Ar/RF/PD/5Ag and *m*Ar/RF/PD/5Ag+S fabric, the number of abrasion cycles is three times higher than that of the *m*Ar fabric after UV irradiation. For the *p*Ar fabric, the number of abrasion cycles does not change after UV exposure. For the modified fabrics, it is almost four times and 18 times higher for *p*Ar/RF/PD/5Ag and *p*Ar/RF/PD/5Ag+S, respectively, compared to the UV irradiated *p*Ar fabrics. For the layer-by-layer method, after UV irradiation, no breakage of the fibers is observed after 100,000 cycles for both types of aramids, as before UV exposure (Figure S1b, Supplementary Materials).

### 3.5. Conductive Properties

**Figure 12:**
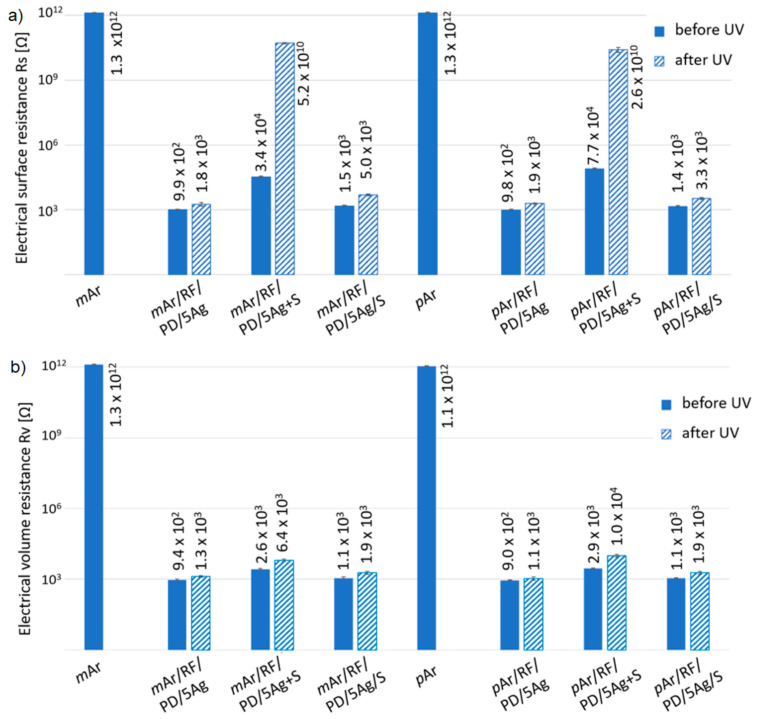
Electrical surface resistance (R_s_) (**a**) and electrical volume resistance (R_v_) (**b**) of unmodified and modified *m*Ar and *p*Ar fabrics before and after UV irradiation (365 nm, 96 h).

The unmodified *m*Ar and *p*Ar fabrics are non-conductive, and their electrical surface resistance (R_s_) is 1.30 × 10^12^ Ω and 1.26 × 10^12^ Ω (Figure 12a), respectively. For both aramid fabrics after the 5-fold AgNWs application, a significant decrease in R_s_ values by 10 orders of magnitude is observed. For both aramid fabrics modified with silanes by the mixture and the layer-by-layer method, a decrease in the R_s_ value by eight and nine orders of magnitude, respectively, is observed compared with the reference fabrics. 

The electrical volume resistance (R_v_) value is 1.30 × 10^12^ Ω and 1.06 × 10^12^ Ω (Figure 12b) for the *m*Ar and *p*Ar fabrics, respectively. After the 5-fold AgNW application, the R_v_ value decreases by 10 orders of magnitude for both fabrics. After the AgNWs and silanes mixture and layer-by-layer modification, the R_v_ values are lower than those of the unmodified fabrics by nine orders of magnitude. 

The slightly higher values of R_s_ and R_v_ for the fabrics modified with silanes by the mixture and layer-by-layer method compared to the fabrics with only AgNWs are due to the presence of silanes, which, on aramid fabrics, have higher R_s_ and R_v_ values of 5.98 × 10^7^ Ω and 3.77 × 10^7^ Ω, respectively, for the *m*Ar fabric and 3.30 × 10^8^ Ω and 6.91 × 10^7^ Ω, respectively, for the *p*Ar fabric. Silanes can be a barrier to the flow of electrons through AgNWs. Moreover, higher values of both resistances are found for the mixture method than for the layer-by-layer method, which may be due to the AgNWs being covered by silanes, which limits their direct connection with each other (which can be seen in the SEM images, Figure 7c and Figure 8c). On the other hand, for the layer-by-layer method, fibers that protrude above the silanes layer are densely covered with AgNWs, which create conductive paths. Under the silanes layer, both on the surface and between the fibers, there are AgNWs that connect with each other, which ensures a lower R_v_ value compared to the fabrics modified with the mixture method.

Moreover, the lower values of R_v_ in relation to R_s_ for all modified fabrics may be due to the AgNWs filling the spaces between the fibers.

After UV irradiation, the R_s_ values increase by one order of magnitude for both the AgNWs modified fabrics (Figure 12a). This increase may be caused by the degradation of AgNWs, which is visible in the SEM images as silver precipitates on the surface of the nanowires (Figure 7b and Figure 8b). For the mixture method, the R_s_ values increase by six orders of magnitude and the fabric becomes non-conductive. For the layer-by-layer method, the R_s_ value has the same order of magnitude. These differences are related to the different thicknesses of the silanes layer. For the layer-by-layer method, the layer is thicker and protects the AgNWs from the UV radiation. In the case of the mixture method, the silanes coating that covers the AgNWs is thin and does not reflect the UV radiation as efficiently. For fabrics modified with the AgNWs and silanes mixture, the coating is discontinuous and damaged with numerous cavities and cracks (Figure 7c and Figure 8c). This causes interruption percolating paths formed by the AgNWs, which prevent the flow of electrons.

The UV radiation causes an increase in the R_v_ values for the AgNWs modified fabrics by one order of magnitude (Figure 12b). For the layer-by-layer method, the value only slightly increases. The R_v_ values of *m*Ar/RF/PD/5Ag+S increase but have the same order of magnitude. For *p*Ar/RF/PD/5Ag+S, an increase by one order of magnitude is observed. The lower increase in R_v_ compared to R_s_ after irradiation is due to the fact that AgNWs located between the fibers inside the fabric are less exposed to UV radiation than the AgNWs on its surface. 

## 4. Conclusions

Multifunctional *meta*- and *para*-aramid fabrics modified with AgNWs and a mixture of silanes, APTES, and DEDMS, were obtained. The aim of the studies was to protect against UV radiation and improve the mechanical, electrical, and hydrophobic properties of modified aramid fabrics. New functionalization methods, a one-step method (mixture) with AgNWs dispersed in the silanes mixture, and a two-step method (layer-by-layer) in which the silanes mixture was applied on the previously deposited AgNWs layer, were developed. 

Fabrics were pre-treated in a low-pressure air RF plasma, and a subsequent polydopamine coating was applied. 

The modified fabrics acquired hydrophobic properties. The water contact angle for the *meta*- and *para*-aramid fabric was 125° and 120°, respectively, for the mixture and 112° and 114°, respectively, for the layer-by-layer method. The surface free energy for the AgNWs and silanes modified fabrics is lower by about 30% for the *meta*-aramid fabric and 50% for the *para*-aramid fabric compared with the reference fabrics.

Better resistance to UV radiation was achieved for the layer-by-layer method. The UV radiation caused no changes in the layer-by-layer coating, while for the mixture coating, there was damage, and some coating fragments were detached. The UV radiation caused a significant decrease in the specific strength of the reference fabrics by about 50% and 60% for the *meta*- and *para*-aramid fabric, respectively, while for the mixture and layer-by-layer method, the specific strength values were 71% and 63% higher for the *meta*-aramid fabric, respectively, and 102% and 110% higher for the *para*-aramid fabric, respectively. The layer-by-layer modified fabrics were the most abrasion resistant; before and after UV radiation, no thread damage was observed after 100,000 abrasion cycles. The electrical surface resistance for the layer-by-layer method was nine orders of magnitude lower than the reference fabrics, and for the mixture method, it was eight orders of magnitude lower. After UV irradiation, the R_s_ did not change for the layer-by-layer method and increased by six orders of magnitude for the mixture modified fabrics, which became non-conductive. We selected the layer-by-layer method as the most effective for aramid fabric modification; it results in great surface, electrical, and mechanical properties and better resistance to UV radiation. It protects aramid fabrics from degradation and the deterioration of their functional properties.

## Figures and Tables

**Table 1:** Mass per unit area of the reference and modified aramid fabrics.

Fabric	Mass per Unit Area, g/m^2^	Mass per Unit Area of AgNW Coating, g/m^2^	Mass per Unit Area of Silanes Coating, g/m^2^	Mass per Unit Area of AgNWs and Silanes Coating, g/m^2^
*m*Ar	205 ± 2			
*m*Ar/RF/PD/1Ag	214 ± 6	9 ± 3		
*m*Ar/RF/PD/5Ag	248 ± 7	43 ± 2		
*m*Ar/RF/PD/5Ag+S	273 ± 7			68 ± 5
*m*Ar/RF/PD/5Ag/S	305 ± 9	43 ± 2	62 ± 1	105 ± 3
*p*Ar	165 ± 3			
*p*Ar/RF/PD/1Ag	171 ± 4	6 ± 1		
*p*Ar/RF/PD/5Ag	200 ± 1	35 ± 3		
*p*Ar/RF/PD/5Ag+S	222 ± 5			57 ± 2
*p*Ar/RF/PD/5Ag/S	263 ± 4	35 ± 3	63 ± 5	98 ± 6

**Table 2:** Characteristics of standard liquids.

Liquid	Surface Tension, mJ/m^2^		
	γ_l_	γ_l_^d^	γ_l_^p^
Water (distilled)	72.8	21.8	51.0
Formamide (99.5%, Sigma-Aldrich, UK)	58.0	39.0	19.0
Diiodomethane (99%, Sigma-Aldrich, UK)	50.8	48.5	2.3

**Table 3:** FTIR bands assigned for the pAr fabric and the reference polydopamine film.

*p*Ar Fabric		Reference Polydopamine Film	
Bands, cm^−1^	Description [7,10,34]	Bands, cm^−1^	Description [7,10,34]
698	C–H out of plane substituted aromatic ring	698	C–H out of plane substituted aromatic ring
821	*p*-substituted phenyl	911	CONH bending, C–N stretching
1306	C–N stretching	1034	C–O stretching
1509	C=C stretching	1113	C–H bending
1538	N–H bending	1335	C–N stretching
1637	C=O stretching	1413	C=O stretching
1740	C=O stretching	1440	C=C stretching vibrations of an aromatic ring
3323	N–H stretching	1527	C=N stretching vibrations of an aromatic ring
		1594	C=C stretching vibrations of an aromatic ring, C–N stretching
		3150	N–H stretching

**Table 4:** The mean values of the weight percentages of the elements with standard deviations for unmodified and modified *m*Ar and *p*Ar fabrics.

Fabric	Weight Percentage of Elements, wt.%				
	C	N	O	Si	Ag
*m*Ar ^1^	68 ± 1	10 ± 1	21 ± 1		
*m*Ar/RF/PD/1Ag ^2^	68 ± 1	8 ± 1	20 ± 1		3 ± 1
*m*Ar/RF/PD/5Ag ^2^	56 ± 2	7 ± 1	18 ± 1		18 ± 2
*m*Ar/RF/PD/5Ag+S ^2^	54 ± 1	6 ± 1	28 ± 1	5 ± 1	6 ± 1
*m*Ar/RF/PD/5Ag/S ^2^	41 ± 1	8 ± 1	31 ± 1	7 ± 1	12 ± 1
*p*Ar ^1^	66 ± 1	13 ± 1	19 ± 1		
*p*Ar/RF/PD/1Ag ^1,2^	66 ± 1	6 ± 1	20 ± 1		6 ± 1
*p*Ar/RF/PD/5Ag ^1,2^	54 ± 3	5 ± 1	18 ± 1		21 ± 3
*p*Ar/RF/PD/5Ag+S ^1,2^	54 ± 1	5 ± 1	26 ± 1	5 ± 1	8 ± 1
*p*Ar/RF/PD/5Ag/S ^1,2^	42 ± 1	7 ± 1	31 ± 1	6 ± 1	12 ± 1

^1^ The total weight percentage of the elements is less than 100 wt.% because of the trace amounts of Na and S (S only for the *p*Ar fabric), which are less than 1 wt.% and are not included in Table 2. In the case of *m*Ar, the presence of Na is due to the technology of fiber production as a result of the reaction of two comonomers in tetrahydrofuran. An oligomer suspension is formed, which combines with sodium carbonate, and fibers are formed [7,25]. In turn, *p*Ar fibers are produced by the condensation of 1,4-diaminobenzene and terephthaloyl chloride. The formed *p*Ar is immersed in sulfuric acid, and then sodium hydroxide is added for neutralization. The product of neutralization is sodium sulphate [7,25]. ^2^ For all AgNWs modified fabrics, the trace amounts of Cl (<1 wt.%), which came from silver chloride as a by-product of the AgNWs colloid synthesis, were present and are not included in Table 2.

**Table 5:** Contact angles of water (Θ_W_), formamide (Θ_F_), and diiodomethane (Θ_DIM_) for unmodified and modified *m*Ar and *p*Ar fabrics.

Fabric	Contact Angle [^o^]		
	Θ_W_	Θ_F_	Θ_DIM_
*m*Ar	64 ± 2	58 ± 2	63 ± 2
*m*Ar/RF	19 ± 3	36 ± 3	27 ± 3
*m*Ar/RF/PD	0 ± 0	36 ± 1	90 ± 0
*m*Ar/RF/PD/1Ag	77 ± 2	74 ± 3	36 ± 4
*m*Ar/RF/PD/5Ag	87 ± 1	90 ± 2	32 ± 3
*m*Ar/RF/PD/5Ag+S	125 ± 5	106 ± 2	55 ± 2
*m*Ar/RF/PD/5Ag/S	112 ± 2	96 ± 2	51 ± 1
*p*Ar	77 ± 4	33 ± 2	14 ± 3
*p*Ar/RF	12 ± 5	18 ± 5	5 ± 1
*p*Ar/RF/PD	0 ± 0	19 ± 1	68 ± 1
*p*Ar/RF/PD/1Ag	84 ± 4	77 ± 2	36 ± 3
*p*Ar/RF/PD/5Ag	89 ± 3	85 ± 2	34 ± 4
*p*Ar/RF/PD/5Ag+S	120 ± 1	106 ± 3	58 ± 2
*p*Ar/RF/PD/5Ag/S	114 ± 2	100 ± 1	53 ± 1

## Data Availability

Not applicable.

## Supplementary Materials

The following supporting information can be downloaded at: https://www.mdpi.com/article/10.3390/molecules27061952/s1, Figure S1: Images of (a) *m*Ar and (b) *p*Ar fabrics surfaces, before and after UV radiation (365 nm, 96 h), before and after abrasion cycles.

## Sample Availability

Samples of the compounds are not available from the authors.
