# Supplementary material for: Silver Nanowires and Silanes in Hybrid Functionalization of Aramid Fabrics
Source: Molecules. 2022 Mar 17;27(6):1952. doi: 10.3390/molecules27061952 (PMC8954008; doi:10.3390/molecules27061952)
Supplement: Supplementary file 1 [file molecules-27-01952-s001.zip › molecules-1629019-supplementary.pdf]

molecules

MDPI

Supplementary Materials

Alicja Nejman [1,2], Anna Baranowska-Korczyc [1], Katarzyna Ranoszek-Soliwoda [2], Izabela Jasińska [1], Grzegorz Celichowski [2] and Małgorzata Cieślak [1,*]

1 ŁUKASIEWICZ Research Network – Textile Research Institute, Brzezinska St. 5/15, 92-103 Lodz, Poland; alicja.nejman@iw.lukasiewicz.gov.pl (A.N.); anna.baranowska-korczyc@iw.lukasiewicz.gov.pl (A.B.-K.); izabela.jasinska@iw.lukasiewicz.gov.pl (I.J.)

2 Department of Materials Technology and Chemistry, Faculty of Chemistry, University of Lodz, Pomorska St. 163, 90-236 Lodz, Poland; katarzyna.soliwoda@chemia.uni.lodz.pl (K.R.-S.), grzegorz.celichowski@chemia.uni.lodz.pl (G.C.)

* Correspondence: malgorzata.cieslak@iw.lukasiewicz.gov.pl (M.C.)

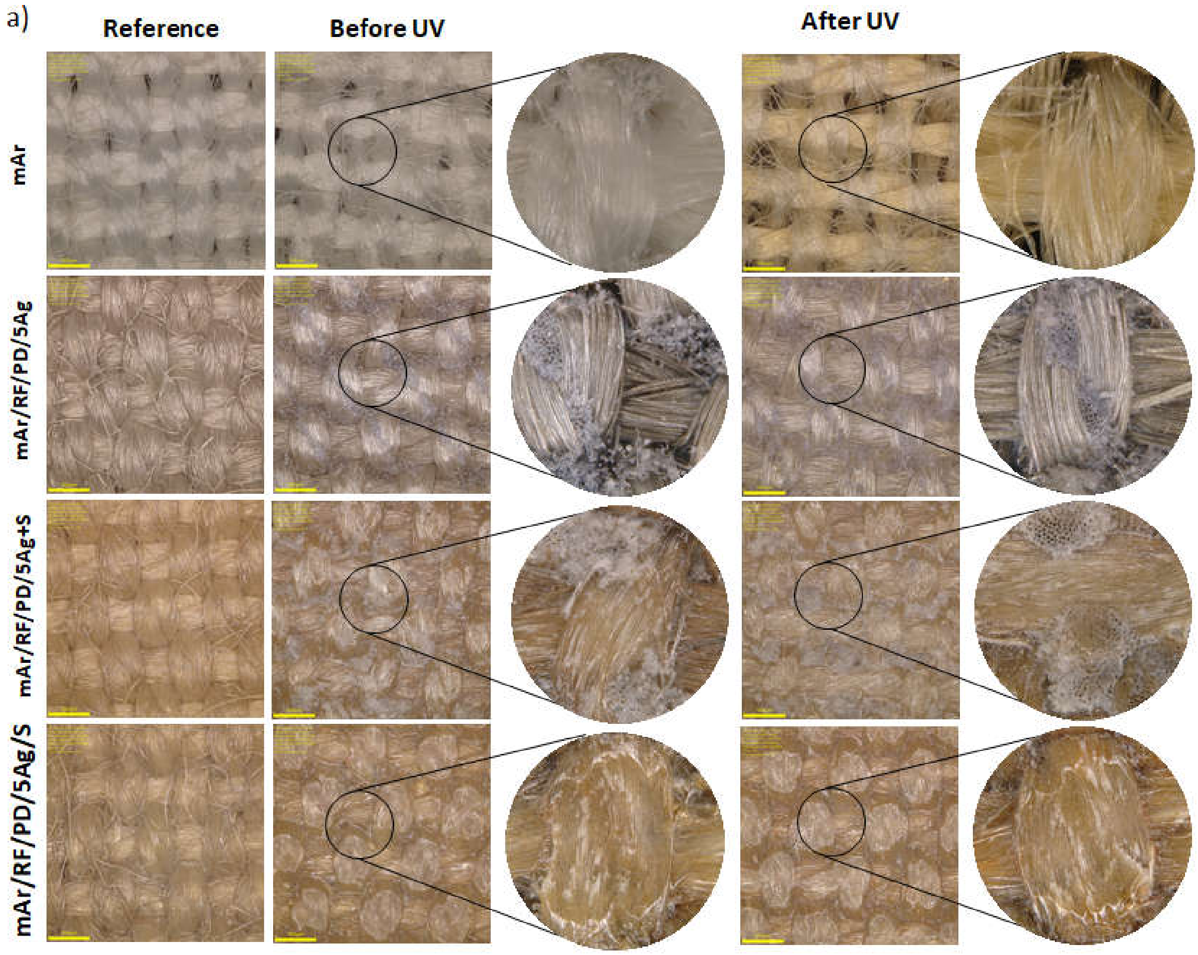

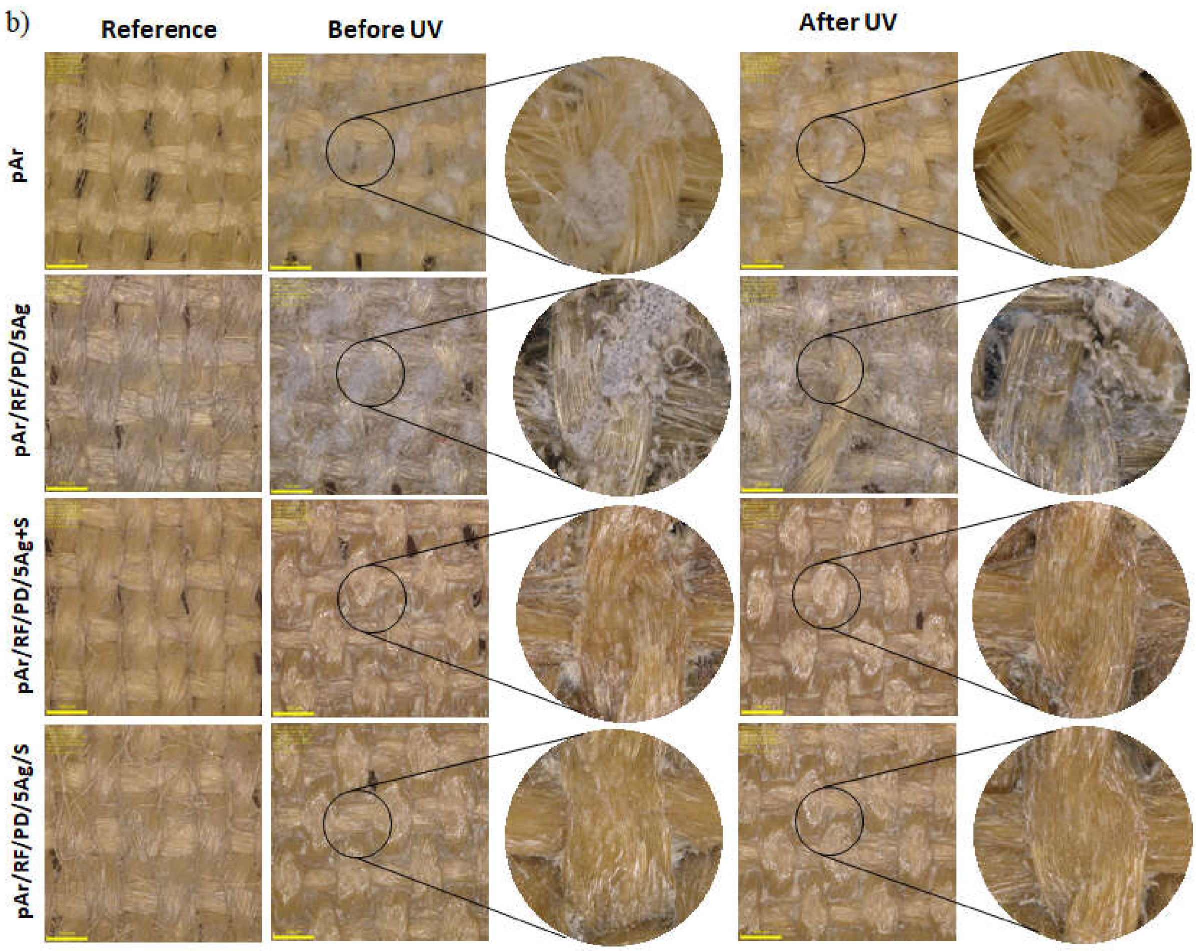

Figure S1. Images of (**a**) *m*Ar and (**b**) *p*Ar fabrics surfaces, before and after UV radiation (365 nm, 96 h), before and after abrasion cycles.
